# Model Systems to Study the Chronic, Polymicrobial Infections in Cystic Fibrosis: Current Approaches and Exploring Future Directions

**DOI:** 10.1128/mBio.01763-21

**Published:** 2021-09-21

**Authors:** George A. O’Toole, Aurélie Crabbé, Rolf Kümmerli, John J. LiPuma, Jennifer M. Bomberger, Jane C. Davies, Dominique Limoli, Vanessa V. Phelan, James B. Bliska, William H. DePas, Lars E. Dietrich, Thomas H. Hampton, Ryan Hunter, Cezar M. Khursigara, Alexa Price-Whelan, Alix Ashare, Robert A. Cramer, Joanna B. Goldberg, Freya Harrison, Deborah A. Hogan, Michael A. Henson, Dean R. Madden, Jared R. Mayers, Carey Nadell, Dianne Newman, Alice Prince, Damian W. Rivett, Joseph D. Schwartzman, Daniel Schultz, Donald C. Sheppard, Alan R. Smyth, Melanie A. Spero, Bruce A. Stanton, Paul E. Turner, Chris van der Gast, Fiona J. Whelan, Rachel Whitaker, Katrine Whiteson

**Affiliations:** a Geisel School of Medicine at Dartmouth, Hanover, New Hampshire, USA; b Ghent University, Ghent, Belgium; c University of Zurich, Zurich, Switzerland; d University of Michigan, Ann Arbor, Michigan, USA; e University of Pittsburgh, Pittburgh, Pennsylvania, USA; f Imperial College London and Royal Brompton Hospital, London, United Kingdom; g University of Iowa, Iowa City, Iowa, USA; h University of Colorado, Anschutz Medical Campus, Aurora, Colorado, USA; i Columbia University, New York, New York, USA; j University of Minnesota, Minneapolis, Minnesota, USA; k University of Guelph, Guelph, Canada; l Emory University School of Medicine, Atlanta, Georgia, USA; m University of Warwick, Coventry, United Kingdom; n University of Massachusetts and the Institute for Applied Life Sciences, Amherst, Massachusetts, USA; o Brigham and Women’s Hospital and Harvard University, Boston, Massachusetts, USA; p Dartmouth College, Hanover, New Hampshire, USA; q Caltech, Pasadena, California, USA; r Manchester Metropolitan University, Manchester, United Kingdom; s McGill University, Montreal, Canada; t University of Nottingham, Nottingham, United Kingdom; u University of Oregon, Eugene, Oregon, USA; v Yale University, New Haven, Connecticut, USA; w University of Illinois, Urbana-Champaign, Illinois, USA; x University of California, Irvine, California, USA; University of Washington

**Keywords:** cystic fibrosis, models, polymicrobial, airway, chronic infection

## Abstract

A recent workshop titled “Developing Models to Study Polymicrobial Infections,” sponsored by the Dartmouth Cystic Fibrosis Center (DartCF), explored the development of new models to study the polymicrobial infections associated with the airways of persons with cystic fibrosis (CF). The workshop gathered 35+ investigators over two virtual sessions. Here, we present the findings of this workshop, summarize some of the challenges involved with developing such models, and suggest three frameworks to tackle this complex problem. The frameworks proposed here, we believe, could be generally useful in developing new model systems for other infectious diseases. Developing and validating new approaches to study the complex polymicrobial communities in the CF airway could open windows to new therapeutics to treat these recalcitrant infections, as well as uncovering organizing principles applicable to chronic polymicrobial infections more generally.

## OPINION/HYPOTHESIS

On 25 to 26 May 2021, over 35 investigators met virtually in a workshop titled “Developing Models to Study Polymicrobial Infections,” sponsored by the Dartmouth Cystic Fibrosis Center (DartCF [https://sites.dartmouth.edu/dartcf/]). The investigators who participated, including 30 Ph.D.s, 6 M.D.s, and an M.D./Ph.D., learned of the meeting via direct email invitation, sharing of the emails, and word of mouth. The initial size of the conference had been limited based on the available funding provided by a grant from the Cystic Fibrosis Foundation; however, the onset of the coronavirus disease 2019 (COVID-19) pandemic and the switch of the workshop to a virtual format allowed us to open the event to a larger and more geographically diverse population. The goal of the workshop was to discuss current model systems for studying polymicrobial infections in the airways of persons with cystic fibrosis (pwCF), to consider their strengths and weaknesses, and to envision how new, improved models could be developed. The workshop was held over 2 days. On each day there were breakout sessions; the goal of each session was to identify 3 to 5 key concepts or ideas, followed by the entire group gathering to discuss the breakout group findings.

## WHAT IS THE GOAL?

Maybe the first question to ask is, “What is the goal of building new and better model systems to study CF-associated polymicrobial infections?” The simple answer is “To help pwCF”; but how? Do we want to determine how to keep microbes from flourishing and what makes them thrive? That is, we could use these new models as drug discovery platforms. Do we want to eradicate the communities or maintain the communities but keep them stable? Are we trying to better understand microbial community function, and do we want to uncover general rules that can be applied to CF-relevant infections, and beyond? Do we want to build predictive models that will help clinicians to know when a pwCF will take a turn for the worse and to intervene earlier and more successfully? And will the new wave of CFTR-targeted therapeutics impact what it means to build effective models? These points and others were addressed during the workshop.

Three overarching themes emerged during the course of the workshop. The first theme is, “What is your question?” This query is an excellent first step in designing any research project, and here, it specifically means that the particular scientific problem driving the investigator will likely dictate which model system they select. That is, in some cases a relatively simple *in vitro* model might suffice, versus a complex animal model. This first question is related to the second, a variation on one of our favorite axioms, “All models are wrong, but some are useful,” often attributed to statistician George Box, but generally applicable across all of science. Here, the phrase can be taken to mean that no model system we develop to study chronic polymicrobial infections in the airways of pwCF will capture the true complexity or spatial/temporal heterogeneity of the CF airway. Finally, and of a practical nature, is the idea that there is not “a” model system to study polymicrobial airway infections for pwCF, because there is not “a” single microbiome associated with this disease. It will almost definitely be the case that a set of models will need to be developed (i.e., a microbial community dominated by one pathogen versus an evenly distributed collection of microbes) to encompass most/all of the variety of CF-associated airway infection communities.

Below we present three frameworks focused on developing models to study polymicrobial airway infections in CF; these frameworks are equally relevant to other infectious diseases. The first framework is to achieve the “perfect” model system(s). The second and third frameworks consider that on the road to the perfect model, there can be the development of model systems which move beyond single-culture experiments in lysogeny broth (LB) but which capture meaningful information of at least key aspects of “reality.” The second framework takes a question-driven approach, and the third considers model systems as hypothesis generators. In this piece, we hope to convince the reader that taking steps on the road toward the perfect model systems is a useful, and we would say critical, exercise in working toward our goal to effectively reduce the infection burden of pwCF. Finally, the very fact that we could gather such an illustrious group of investigators from around the United States and the world in one (virtual) place and at one time speaks to the dedication and engagement of the larger CF research community toward this goal.

## FRAMEWORK 1: WORKING TOWARD THE PERFECT MODEL SYSTEM(S)

Ideally, researchers would have access to a set of “do-it-all” model systems that capture much/all of the complexity of the *in vivo* environment. This is a lofty goal, and this approach will get very complex, very quickly. Here, we put practicality aside and discuss the factors we should consider when building “perfect” model systems.

As part of this framework, it is critical to note upfront that CF is not a singular disease or pathological condition. Rather, as a chronic, lifelong (and life-shortening) condition, its features (airway microbiota, airway environment, and host) are constantly changing and evolving. To model polymicrobial infection in CF, we need to first ask, “what CF disease are we trying to model?” In the short term, pwCF cycle between periods of relative clinical stability, punctuated by intermittent, unpredictable episodes of poorer health (i.e., exacerbations) that may reflect acute changes in the airway microbiota, environment, and/or host. Over longer periods, as CF lung disease inevitably progresses, the airway environment changes with concomitant changes in community composition and activity, which can be (admittedly arbitrarily) defined as disease stage: early, intermediate, and advanced. Finally, aggressiveness describes the pace of disease progression, which seems to be an innate feature of the host, who can have a mild, moderate, or severe phenotype. Of interest, this phenotype is only roughly related to CFTR genotype, and the etiologic underpinnings are unknown but could include modifier genes. And of course, disease phenotype may have a bearing on airway microbial community composition and activity, and vice versa. So, in conclusion, one must consider “which CF” to model: exacerbating versus stable? Early (pediatric) versus advanced (adult) disease? Mild phenotype versus severe phenotype?

### Exploring heterogeneity across time and space.

It is clear that the CF lung is a heterogeneous environment across many dimensions, complicating our understanding of the *in vivo* environment. It is important to note that any heterogeneity in the environment may impact the microbe(s), the host (including the immune response), or the host-microbe(s) interactions, adding yet another layer of complexity. Among the issues one must consider are (i) the right spatial scale to study (bulk, single cell, or somewhere in the middle; micrometers versus nanometers); (ii) the right time scale for studying communities (minutes, hours, days, or years) and whether these infection communities are stable or unstable over time; (iii) how microbial growth rates vary across the airway; (iv) local versus distance effects (cell-cell contact, diffusion of metabolites, proteases, and virulence factors); (v) density of microbes across the airway (i.e., proximal/distal airway, across mucus plugs); (vi) the definition of “chronic,” whether it is the same for all pathogens, and what “chronic” infection is in the context of a model system (perhaps achieving stability of the microbial community or, in an animal model, some indicator of host tissue damage or inflammatory markers or morphological evidence); (vii) how antibiotic tolerance varies across the environment; (viii) how the environment varies with host genotype; (ix) whether there is variation in available electron acceptors (e.g., oxygen, nitrate); (x) how much genetic and/or phenotypic variation exists within a species in a single lung and how many isolates need to be sequenced/analyzed to appreciate such heterogeneity; (xi) the rate of Pseudomonas aeruginosa evolution (given the evidence that isolates vary over time) and whether there are rules that govern the evolutionary trajectory of different pathogens; and finally (xii) how much of the heterogeneity is “real” and how much is sampling error.

### Documenting the *in vivo* environment.

A critical first step in developing a model system is filling the key knowledge gap of understanding the nature of the environment to be modeled, in this case, the CF airway. Documenting this environment involves the following. (i) First is measuring oxygen, pH, viscosity, nutrients (amino acids, lipids, mucus, and eDNA) and micronutrients (metals and their various redox states, which change with changing oxygen availability, for example). Importantly, the spatiotemporal variability of these parameters needs to be characterized. In the case of nutrients, they may be host or microbe derived, and when one can measure these compounds, concentrations might reflect bulk measurements or an average across patchy concentrations or a gradient. (ii) Next is exploring whether the environment is static or characterized by flow, or if there is mixing. (iii) Third is visualizing the microbial communities and their surroundings using approaches like Microbial Identification After Passive Clarity Technique (MiPACT), combinatorial labeling and spectral imaging-fluorescence *in situ* hybridization (CLASI-FISH), or bioorthogonal noncanonical amino acid tagging (BONCAT) (1–3) to appreciate their biogeography. Are the microbes in mixed communities or isolated, and if they are isolated, is cross-diffusion of metabolites and other secreted molecules possible? (iv) Last is appreciating that all of the above parameters likely change as the community evolves, which in turn changes the infection environment, and so on. A clear challenge is how one would make these measurements, as removing samples from the airway of a pwCF and analyzing them invariably causes perturbations. One indirect method to understand the *in situ* environment is to analyze the microbes and their *in vivo* state and infer the environment from the response of the microbes (i.e., use microbes as biosensors). Alternatively, developing or introducing new biological, chemical, or mechanical sensors might be a necessity to document the *in vivo* environment accurately. A significant challenge to this approach is that even if one had all possible information, it is likely that we would not really understand which components of this information are actually important or relevant to CF airway infections. That is, what do we mean when we ask, “What matters?” Using modeling or statistical approaches with data combined across many patients may allow us to differentiate between factors that have a high versus low impact on infections and infection dynamics.

### Contributions of microbes, host, and interactions among these actors.

A model system needs to capture three key factors driving the local infection environment—the contributions of the microbe(s), the host, and the host-microbe interactions. The host-microbe interactions include core microbial metabolisms and virulence factors, as well as the host’s defensive countermeasures. And while the focus of study is often on bacteria, fungi and eukaryotic/bacterial viruses are also key players, and as mentioned above, even within a single species, genetic and/or phenotypic variability can be substantial. Finally, the nature of the host immune response evoked by these organisms, which is highly dependent upon the microbes’ expressed phenotype at various stages of infection, is also an important variable that must be considered.

### Design factors for model communities.

As described above, it is likely that more than one infection community would be needed to effectively capture the variety of CF infections. How does one decide on such communities? Approaches to identify communities could focus on (i) infection severity (mild, medium, or severe); (ii) clustering common community types based on 16S and/or 18S rRNA amplicon library sequence or metagenomic data, metabolic potential, or other factors; (iii) leveraging an ecological framework by considering stable/unstable communities, community succession, transition from one stable state to the next, or energy conservation as a driver of overall community organization; (iv) establishing model communities with a dominant pathogen versus an “even” distribution; or (v) using abundance versus prevalence data. The challenge here is that some microbes are common across many pwCF (Pseudomonas, Staphylococcus, Streptococcus, and *Prevotella*) while other pathogens are abundant in a few patients but deadly in this context (i.e., *Burkholderia* and the mold Aspergillus fumigatus). *A priori*, it is not obvious which of these community types should be analyzed to maximize *in vivo* relevance in the context of CF. Moreover, how contemporary CFTR-targeted therapies will impact microbial communities is an open question.

### Validating the models.

Any model development would require validation, engaging clinicians, microbiologists, immunologists, and researchers from other fields to help with this process. There are multiple approaches to validating a model, including but not limited to (i) using transcriptomics, proteomics, single-cell studies, or related omics approaches to compare the model to *in vivo* samples; (ii) using phenotypic assays, including antibiotic tolerance profiles or breath condensate profiles, to compare the model to *in vivo* samples; (iii) utilizing validated biomarkers, as they become available, to compare the model to *in vivo* samples; (iv) developing a quantitative accuracy score measure to help compare one model to another and to the *in vivo* setting; (v) assessing whether the model reproduces across labs; and (vi) engaging a team strategy where different labs leverage their specific expertise to validate relevant aspects of the model. Whatever metrics are used, validation should engage multiple lines of evidence and falsifiability.

## FRAMEWORK 2: THE RIGHT MODEL FOR THE QUESTION

Here, the concept is that a particular model might be suited to address a specific question, but the same model might have less utility in other contexts. The issues outlined in framework 1 would need to be considered, but these issues may be much more easily addressed when only a specific aspect of model utility needs to be tackled. A list of models and their current or potential future utility is presented in Table 1; due to lack of space, only a few selected models are briefly discussed here to illustrate the concept underlying this framework.

**TABLE 1:** Utility of model systems for airway infection in CF

Model type	System	Use^*a*^							
		Studies of airway infection	Chronic airway infection	Gut-lung axis studies	General infection studies^*b*^	Studies of basic microbial biology	Studies of host response	New antibiotic discovery	New antibiotic validation
CF animal models	Mouse	Y	Y^*c*^	Y		Y	Y	Y	
	Rat	Y		Y		Y	Y	Y	
	Piglet	Y	F	Y		Y	Y	Y	
	Ferret	Y	F	Y		Y	Y	Y	
	Rabbit	Y				Y	Y	Y	
									
Other animal models	Zebra fish				Y	Y	Y		
	Caenorhabditis elegans				Y	Y	Y		
	Wax moth larvae				Y	Y	Y		

*Ex vivo* models^*d*^	Porcine lung model	Y	to 21 days		Y	Y	Y		Y
	Piglet trachea	F			F	F	F	F	
	Human lung tissue	Y	F		Y	Y	Y		Y
	Human cell lines	Y	F		Y	Y	Y	Y	
	Human primary cells	Y	F				Y		Y
	Organoid-derived 2D cultures	Y	F		Y	Y	Y		Y
	Human lung on chips	Y	F		Y	Y	Y		Y
	Sputum	Y	F		Y	Y	Y	Y	Y

*In vitro* models	LB (standard lab medium)					Y			
	Synthetic CF sputum medium	Y			Y	Y		Y	Y
	Conditioned medium from host cells	Y	F		Y	Y	Y	Y	Y
	Conditioned bacterial supernatant	Y			Y	Y			
	Microfluidic chambers	F^*e*^			F	Y			Y
	WinCF	Y	F		Y	Y			Y
	Chemostats	Y	Y		Y	Y			Y
	Multiwell plates^*f*^	Y	F			Y		Y	

*In silico* models	Bioinformatics and modelling of existing data^*g*^	Y	Y	Y	Y	Y	Y	Y	
	Empirical machine learning models	Y	Y	Y	Y	Y	Y	Y	
	Microbial metabolic models	Y	Y	Y	Y	Y	Y	Y	
	Airway transport models	Y	Y	Y	Y	Y	Y	Y	
	Agent-based models	Y	Y	Y	Y	Y	Y	Y	

Immune system models^*h*^	Immune cells (neutrophils, monocytes, etc.)	Y			Y	Y	Y	Y	Y
	Primary mouse immune cells	Y			Y	Y	Y		Y
	Primary human immune cells^*i*^	Y			Y	Y	Y		Y
	Antibodies	Y	Y	Y	Y	Y	Y		

a Y, yes; F, future development of this use is likely.

b Work that is not airway specific but can help understand general virulence factors or their mechanism of action.

c Agar bead model.

d Classifying *ex vivo* versus *in vitro* models can sometimes be difficult. Here, we classify models as *ex vivo* if they comprise complex tissues or human samples and use primary cells.

e If adapted to use (artificial) sputum.

f Other *in vitro* biofilm models are also available (e.g., Calgary device, beads, tube biofilms, etc.).

g Microbiome, metagenome, transcriptome, proteomes, chemical dynamics, fluid flow, etc., alone or in combination with clinical metadata.

h See also animal and *ex vivo* models.

i Including peripheral and alveolar cells.

### Bacterium-host cell coculture models.

Bacterium-host cell coculture models have been validated (4) and effectively used to study processes including iron cross feeding from host cell to microbe (5), identification and analysis of virulence factors (6, 7), formation of biofilms and the associated high-level biofilm antibiotic tolerance (8, 9), and bacterial/viral/host interactions (10).

### ASM.

The various formulations of artificial sputum medium (ASM) provide excellent (11, 12), and now validated (4), model media for assessing bacterial physiology in a nutrient environment reflective of the CF airway. However, this medium lacks a variety of host-derived metabolites and likely does not reflect the variation in pH, viscosity, or mucin/eDNA concentrations found *in vivo*.

### WinCF.

The WinCF system uses the concept of a Winogradsky column, combined with ASM, to generate gradients (oxygen, pH, and nutrients), with the potential to add additional complexity, such as host metabolites (13). Again, while an improvement over using LB liquid cultures, this system does lack, for example, immune cells and the ability to assess the infections over long time frames.

## FRAMEWORK 3: MODEL SYSTEMS AS HYPOTHESIS GENERATORS

**FIG 1:**
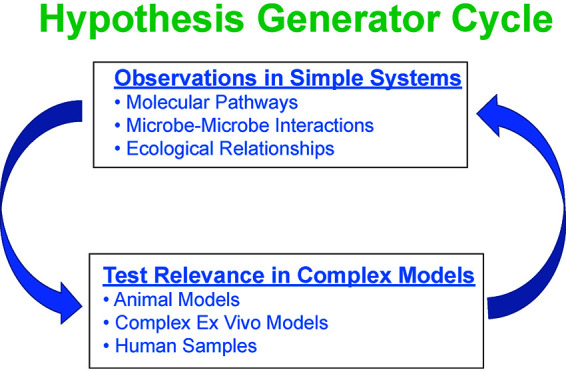
Hypothesis generator cycle. The cycle starts with existing clinical data sets (i.e., 16S rRNA gene amplicon sequences or metagenomes) combined with statistical analyses or predictive modeling to inform the development of relatively simple *in vitro* or *ex vivo* models. Such models are used to learn aspects of molecular mechanisms or ecological principles driving microbe-microbe or host-microbe interactions; the data are then used to drive the next round of hypotheses that can be tested in more complex models or human samples. This process can be repeated multiple times, with each turn of the cycle providing additional insight into the complex polymicrobial infections in CF.

The driving concept here is to develop relatively simple models, based on the best information currently available, and use these models to explore mechanistic details of microbe-microbe or microbe-host interactions. Once a specific mechanistic interaction is dissected, one then has a specific question to answer via exploiting the more complex model systems or human clinical samples (Fig. 1). This bottom-up approach is attractive because it does not rely on a complete understanding of the infection environment before embarking on a study. The danger is the difficulty of starting from a low-level model and predicting what will happen with increasing complexity. The other obvious risk, of course, is that one might discover a biologically interesting phenomenon with no relevance to infection.

A simple polymicrobial model could start with 4 to 6 microbes; approaches for selecting microbes may include modeling of 16S and/or 18S rRNA gene amplicon data sets or mining transcriptomes with tools like ADAGE (analysis using denoising autoencoders of gene expression) (14) (additional considerations are presented in framework 1). The underlying premise is that the infection environment selects for stable communities (pwCF show stable airway communities for often extended periods [15–17]), and analyses of clinical data reveal the composition and features of such communities. It might also be possible to evolve stable communities *in vitro*. Alternatively, one could slowly build complexity—start with early colonizers of the CF airway like Staphylococcus aureus and Haemophilus influenzae, then add a second microbe, a third, and a fourth, and then alter the environment (add mucus, antibiotics, or immune cells), followed by using phenotypic or omics analyses to ask when (if) the community reaches an equilibrium. With any strategy, using reference strains, with all the tools available to study such strains, is an excellent start, which would then need to be supplemented with testing of multiple clinical isolates of each genus, multiple species, and the variety of genotypes that evolve *in vivo* (i.e., *lasR* mutants, nonmotile strains, and antibiotic resistance). Using the well-characterized ASM would be a reasonable place to start for a growth medium. A clear advantage of such an approach is the ability to replicate findings across labs and perhaps even foster the adoption of such systems (with standard strains and media) across multiple labs, generating a shared model system attacked by multiple researchers using their best tools. Adopting common model systems for polymicrobial communities could be akin to labs focusing on Escherichia coli, Bacillus subtilis, Candida albicans, and P. aeruginosa as single-organism models, which helped coalesce efforts and drive progress in understanding basic microbial biology.

### Test hypotheses in more complex systems, including with animal models or human clinical samples.

The idea here is to use the simple models to generate hypotheses to test in more complex systems. Hypotheses generated could be quite focused and assist in the *a priori* selection of the “right model for the job.” Framework 3 should be considered an iterative series of hypothesis-generating experiments, followed by testing the hypotheses in more complex systems (i.e., animal models and clinical samples), followed by improvement of the simple model and exploration to generate the next round of hypotheses to be tested.

## IT IS WELL WORTH THE EFFORT

Developing new and better model systems for CF is a clear and important challenge, but the benefits could be large for pwCF in terms of understanding infection biology and host responses to these infections, as well as developing discovery platforms for new, more efficacious antimicrobial agents. Despite the complexity of recapitulating CF infections *in vitro*, *ex vivo*, or in animals, a concerted effort toward improving model systems raises hope for the emergence of some general organizing principles applicable to chronic infections in CF. Finally, the general frameworks outlined here may provide a strategy for tackling other polymicrobial infections (e.g., chronic wounds and non-CF bronchiectasis), in terms of both studying community structure/function and developing more efficacious therapies.
